# Relative CT Number as a Quantitative Imaging Biomarker for Differentiating Sinonasal Polyp, Inverted Papilloma, and Squamous Cell Carcinoma: A Preliminary Retrospective Study

**DOI:** 10.3390/tomography12100151

**Published:** 2026-10-14

**Authors:** Tuğba Öztürk Taşkıran, Servet Akyüz, Göksel Tuzcu

**Affiliations:** 1 Department of Radiology, Patnos State Hospital, 04500 Ağrı, Türkiye; md.tugbaozturk@gmail.com; 2 Department of Otorhinolaryngology, Faculty of Medicine, Aydın Adnan Menderes University, 09100 Aydın, Türkiye; akyuzservet@gmail.com; 3 Department of Radiology, Faculty of Medicine, Aydın Adnan Menderes University, 09100 Aydın, Türkiye

**Keywords:** sinonasal polyp, inverted papilloma, squamous cell carcinoma, relative CT number, Hounsfield Unit, paranasal sinus, differential diagnosis

## Abstract

**Background/Objectives**: Unilateral sinonasal lesions may be difficult to characterize on non-contrast computed tomography (CT) because inflammatory polyps, benign neoplasms, and malignant tumors can appear as similar unilateral soft-tissue masses. Non-contrast CT is widely available, but the diagnostic performance of relative CT number across inflammatory, benign neoplastic, and malignant sinonasal lesions within a single cohort remains insufficiently characterized. This study aimed to provide additional evidence regarding the diagnostic performance of relative CT number on non-contrast CT by comparing its values among sinonasal polyp (SP), inverted papilloma (IP), and squamous cell carcinoma (SCC), and by evaluating predefined binary diagnostic comparisons. **Methods**: This retrospective single-center study included 92 patients with histopathologically confirmed unilateral or unilateral-dominant sinonasal lesions: 49 SP, 24 IP, and 19 SCC. Preoperative non-contrast paranasal sinus CT images were reviewed. Relative CT number was calculated as the ratio of mean lesion attenuation to mean pontine attenuation. Group differences were analyzed using non-parametric tests, and diagnostic performance was assessed with receiver operating characteristic (ROC) curve analysis. Age-adjusted binary logistic regression analyses were also performed for the predefined binary comparisons. Sensitivity analyses were additionally adjusted for lesion ROI area. **Results**: Relative CT number differed significantly among the three groups (overall *p* < 0.001). ROC analysis showed an AUC of 0.815 (95% CI, 0.704–0.904) for SP versus IP, 0.667 (95% CI, 0.498–0.826) for IP versus SCC, 0.869 (95% CI, 0.792–0.932) for SP versus IP+SCC, and 0.937 (95% CI, 0.866–0.986) for SP versus SCC. After age adjustment, relative CT number was associated with the positive diagnostic category in all four comparisons; however, the IP–SCC association was no longer significant after ROI-area adjustment (*p* = 0.073). The highest diagnostic performance was observed for distinguishing SP from SCC. **Conclusions**: These preliminary findings suggest that relative CT number may serve as an adjunctive quantitative CT parameter for differentiating unilateral inflammatory sinonasal lesions from neoplastic lesions, with the strongest diagnostic performance observed for SP versus SCC. However, its standalone discrimination between IP and SCC was limited, and the age-adjusted association remains hypothesis-generating.

## Simple Summary

Growths affecting one side of the nose and sinuses can look similar on scans despite requiring different treatments. We reviewed scans from 92 patients to assess whether comparing the density of a growth with that of the brainstem could help distinguish inflammatory polyps, benign tumors, and cancer. This measurement showed promise for distinguishing inflammatory polyps from tumors, particularly cancer, but was less useful for separating benign tumors from cancer. It may complement routine scan interpretation, but larger studies are needed before it can guide clinical decisions.

## 1. Introduction

Unilateral sinonasal masses pose a considerable diagnostic challenge, as inflammatory lesions, benign neoplasms, and malignant tumors may present with overlapping symptoms, similar endoscopic appearances, and nonspecific radiological findings [1,2,3]. Sinonasal polyps (SP), the most common benign inflammatory lesion of the sinonasal cavity, are typically bilateral and arise in the context of chronic rhinosinusitis or allergic mucosal inflammation; however, unilateral involvement raises suspicion for alternative diagnoses, including neoplastic pathology [1,2]. Some investigators have proposed that unilateral sinonasal lesions—particularly those presenting as a visible mass or polypoid lesion on endoscopy—should be regarded as neoplastic until proven otherwise [3]. Supporting this view, a 15-year retrospective review of unilateral sinus disease found that, among patients presenting with a unilateral nasal mass or polyp, inverted papilloma (IP) and malignant tumors accounted for approximately 15.7% and 13.7% of cases, respectively, underscoring the importance of reliable preoperative differential diagnosis in this clinical setting [4].

Among unilateral sinonasal tumors, IP occupies a distinct position in the differential diagnosis. Despite its histologically benign nature, IP is characterized by locally aggressive behavior, a high tendency for recurrence, and the risk of malignant transformation—most commonly to squamous cell carcinoma (SCC) [5]. Reported rates of malignant transformation generally range from 5% to 13% [5,6], a variation likely attributable to differences in follow-up duration, study population, and histopathological sampling across studies. This biological profile has direct therapeutic implications. While SP can often be managed with medical treatment or limited endoscopic intervention, IP may require wider resection of the involved mucosa and underlying bone to reduce the risk of recurrence [6]. SCC, in turn, frequently necessitates more aggressive surgical resection and adjuvant therapy. Accurate pretreatment differentiation may support decisions regarding biopsy, surgical extent, multidisciplinary assessment, and adjuvant treatment planning, while reducing the risk of unnecessary aggressive intervention for benign inflammatory lesions.

Computed tomography (CT) and magnetic resonance imaging (MRI) have complementary roles in the evaluation of sinonasal lesions. MRI, particularly contrast-enhanced MRI, provides superior soft-tissue characterization and may facilitate differentiation between solid tumor components and retained inflammatory secretions. Non-contrast CT, however, is rapid, widely available, and provides excellent assessment of sinonasal anatomy and osseous changes, including remodeling, focal hyperostosis, cortical erosion, and bone destruction. Nevertheless, on non-contrast CT, sinonasal polyps, inverted papillomas, and squamous cell carcinomas may appear as similar unilateral soft-tissue masses, limiting the specificity of qualitative CT assessment [7,8].

Low-dose CT and cone-beam CT have also been investigated for sinonasal anatomical assessment [9]. However, reduced-dose CT protocols may compromise the visibility of fine anatomical structures, including the lamina papyracea and cribriform plate, particularly in patients with extensive polyposis or previous sinus surgery [10]. Nasal endoscopy may help guide patient selection for low-dose CT by assessing the extent of polyposis [11]. The impact of reduced-dose protocols on quantitative attenuation measurements such as relative CT number, however, requires further evaluation. The present study used a single institutional non-contrast CT protocol.

To address the difficulty of differentiating these lesions on CT, several studies have explored radiomics, machine learning, and advanced computational imaging approaches [12,13]. Alongside these complex methodologies, relative CT number, defined as lesion attenuation normalized to pontine attenuation, has been proposed as a simpler and more interpretable CT-derived quantitative parameter. The pons was selected as an internal reference structure outside the sinonasal region, following the approach used by Azuma et al. and Sano et al. [14,15]. Normalization to pontine attenuation is intended to reduce variability related to scanner calibration. Sano et al. reported that a relative CT number > 1.3 differentiated IP from nasal polyps with an AUC of 0.82 (sensitivity, 72.6%; specificity, 87.3%) in a cohort of 122 patients [15], while Azuma et al. demonstrated that a relative CT number ≥ 1.4 differentiated SCC from IP with an AUC of 0.77 (sensitivity, 72%; specificity, 83%) in 54 patients [14]. However, neither study evaluated all three lesion types within a single cohort, and the extent to which these two independently derived cut-off values (1.3 and 1.4) remain valid or comparable when SP, IP, and SCC are assessed together has not been established. Therefore, whether relative CT number demonstrates consistent differences among SP, IP, and SCC when evaluated within a single cohort, and how effectively it distinguishes inflammatory lesions from neoplastic lesions, remains insufficiently characterized.

We hypothesized that relative CT number would increase progressively across the inflammatory-to-malignant spectrum, with the lowest values in SP, intermediate values in IP, and the highest values in SCC, reflecting the increasing tissue density, vascularity, and cellularity associated with neoplastic and malignant transformation. SP, IP, and SCC were selected to represent inflammatory, benign neoplastic, and malignant lesions in the differential diagnosis of unilateral sinonasal masses. Accordingly, this study aimed to compare relative CT number values across SP, IP, and SCC groups, and to evaluate its diagnostic performance across clinically meaningful binary comparisons, including the differentiation of inflammatory from neoplastic lesions.

## 2. Materials and Methods

### 2.1. Study Design

This retrospective, single-center observational study was conducted at Aydın Adnan Menderes University Faculty of Medicine Hospital. Patients who received a histopathologically confirmed diagnosis of SP, IP, or SCC between December 2021 and December 2024, and who had undergone preoperative non-contrast paranasal sinus CT, were eligible for inclusion.

### 2.2. Ethical Approval

The study protocol was approved by the Non-Interventional Clinical Research Ethics Committee of Aydın Adnan Menderes University Faculty of Medicine (Approval No: E-53043469-050.04-723942; Date: 29 April 2025) and was conducted in accordance with the principles of the Declaration of Helsinki. Informed consent was waived owing to the retrospective nature of the study.

### 2.3. Inclusion and Exclusion Criteria

Patients were included if they had: (1) a histopathologically confirmed diagnosis of SP, IP, or SCC; (2) a preoperative non-contrast paranasal sinus CT examination performed before surgical treatment; (3) a unilateral or predominantly unilateral sinonasal lesion on CT; and (4) sufficient image quality to allow reliable region of interest (ROI) placement within the lesion. The presence or absence of bone erosion/invasion was not a criterion for patient selection. Lesions confined to the sinonasal cavities and those extending beyond them were eligible, provided that the other inclusion criteria were met.

**Figure 1:**
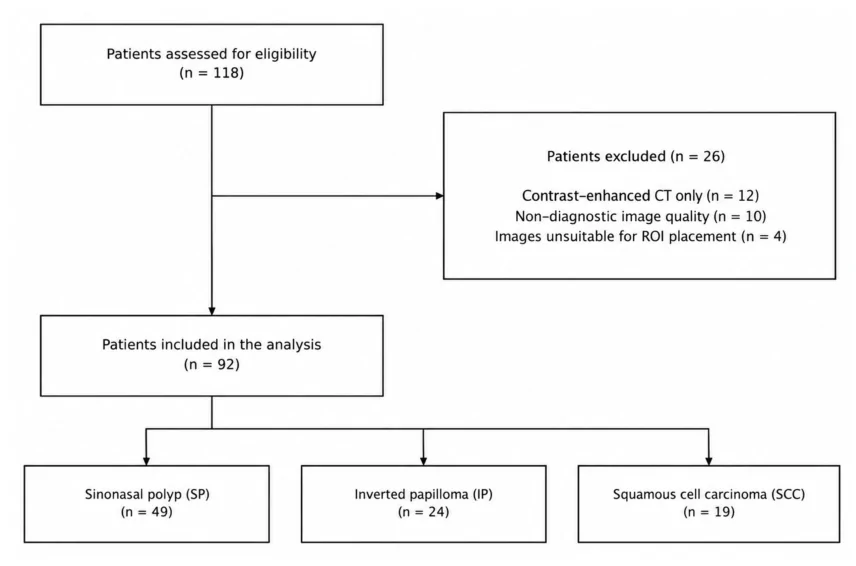
Flowchart of patient selection. ROI, region of interest; CT, computed tomography; SP, sinonasal polyp; IP, inverted papilloma; SCC, squamous cell carcinoma.

Patients were excluded on the following grounds: repeat imaging from the same episode or recurrent disease; availability of contrast-enhanced CT only; non-diagnostic image quality due to motion or metal artifact; extensive necrosis or marked heterogeneity precluding reliable ROI placement; and incomplete clinical records. A total of 118 patients were identified through a search of the institutional pathology database for histopathologically confirmed SP, IP, or SCC diagnosed between December 2021 and December 2024. Of the 118 patients assessed for eligibility, 26 were excluded: 12 had only contrast-enhanced CT examinations, 10 had inadequate image quality, and four had images unsuitable for ROI placement. The final cohort comprised 92 patients: 49 with SP, 24 with IP, and 19 with SCC. The patient selection process is summarized in Figure 1.

### 2.4. Clinical and Demographic Data

Patient demographics and clinical data, including age, sex, and side of lesion, were retrospectively retrieved from medical records. Imaging findings recorded for each patient included lesion attenuation, lesion ROI area, pontine attenuation, and the presence and pattern of adjacent bone changes, as detailed in the following sections.

### 2.5. CT Image Analysis

All non-contrast paranasal sinus CT examinations were performed using a 160-slice, 128-detector multidetector computed tomography scanner (Aquilion Prime, Toshiba Medical Systems, Otawara, Japan). Acquisition parameters included a tube voltage of 120 kVp with automatic tube current modulation (approximately 100–250 mAs). Images were acquired in the axial plane and reconstructed in coronal and sagittal planes using both bone and soft-tissue reconstruction algorithms, with a slice thickness and reconstruction interval of 3 mm, in accordance with the institutional imaging protocol. Lesion and pontine attenuation measurements were performed on soft-tissue kernel images, whereas adjacent bone changes were assessed on bone kernel images. All images were retrospectively retrieved from the institutional picture archiving and communication system and analyzed using Sectra IDS7 (Sectra AB, Linköping, Sweden).

All quantitative assessments were performed on preoperative non-contrast paranasal sinus CT images. Only cases with unilateral sinonasal masses or lesions with clear unilateral predominance—representing a genuine differential diagnostic challenge—were included in the analysis. Image analysis, including ROI placement and attenuation measurements, was performed by a radiologist with five years of experience in head and neck radiology, who was blinded to clinical presentation, endoscopic findings, and histopathological diagnoses. All images and measurements were subsequently reviewed jointly by both radiologists, who remained blinded to histopathological diagnoses, and the final measurements were agreed upon by consensus. Parameters recorded for each case included lesion attenuation, lesion ROI area, pontine attenuation, and the presence and pattern of adjacent bone changes.

**Figure 2:**
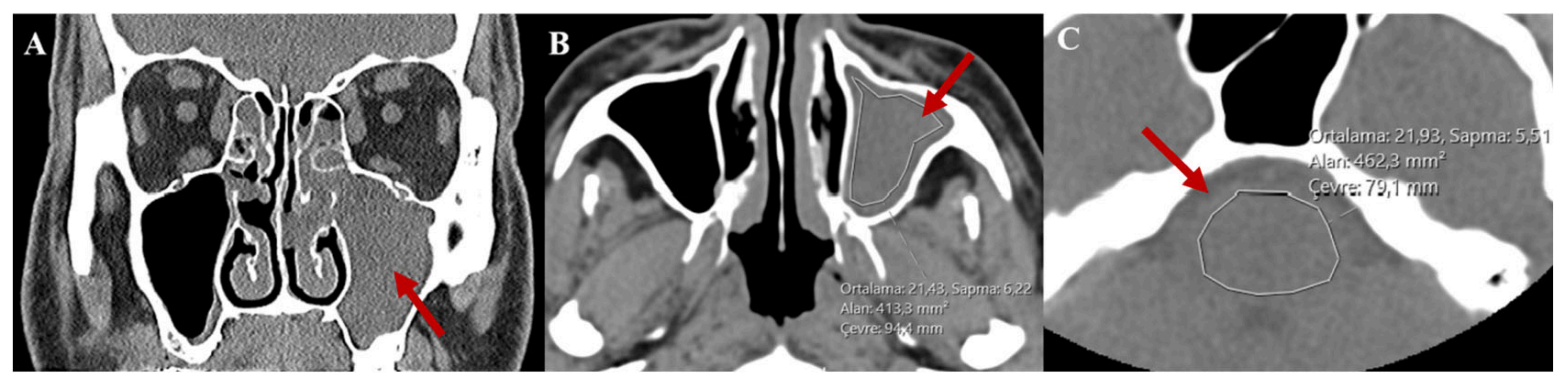
A 45-year-old male patient diagnosed with SP. (**A**) Soft tissue mass observed in the left maxillary sinus (arrow). (**B**) The mean attenuation of the lesion was measured as 21.43 HU, and (**C**) the mean attenuation of the pons was measured as 21.93 HU. Arrows in panels (**B**,**C**) indicate the ROI boundaries. The relative CT number was calculated as 0.97.

**Figure 3:**
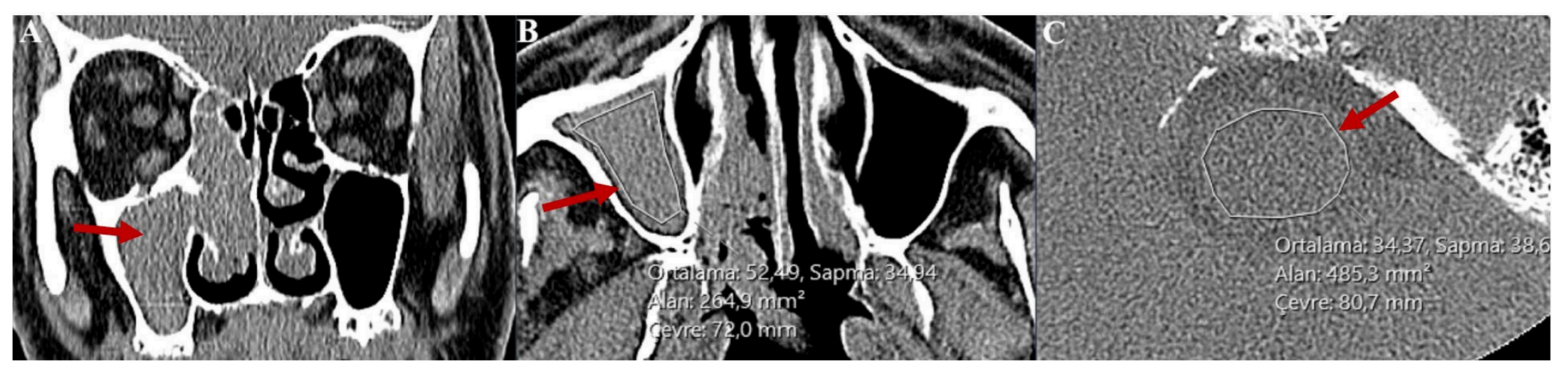
A 70-year-old male patient diagnosed with IP. (**A**) Soft tissue mass observed in the right maxillary sinus (arrow). (**B**) The mean attenuation of the lesion was measured as 52.49 HU, and (**C**) the mean attenuation of the pons was measured as 34.37 HU. Arrows in panels (**B**,**C**) indicate the ROI boundaries. The relative CT number was calculated as 1.52.

**Figure 4:**
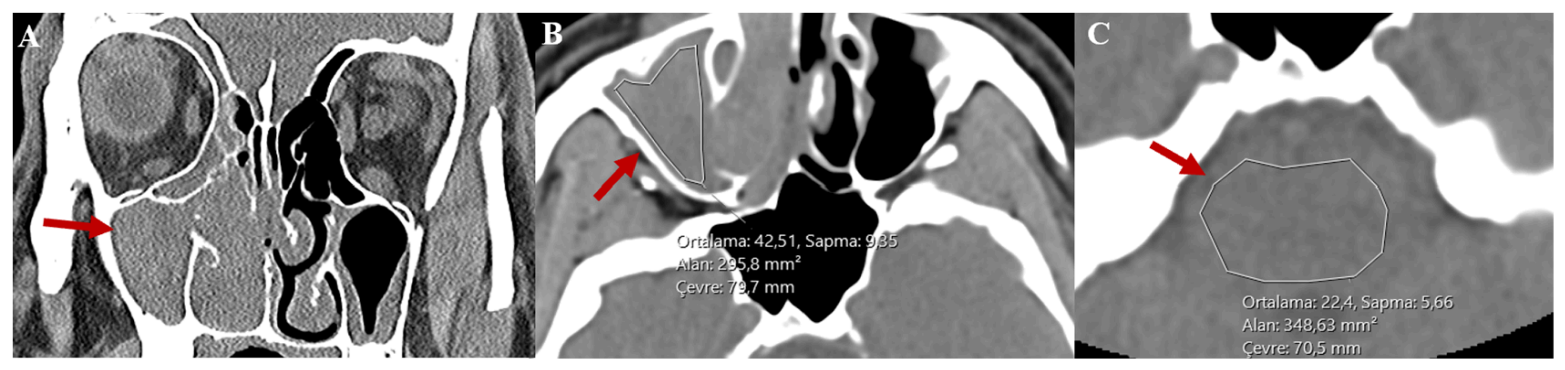
A 50-year-old male patient diagnosed with SCC. (**A**) Soft tissue mass observed in the right maxillary sinus extending into the adjacent nasal cavity (arrow). (**B**) The mean attenuation of the lesion was measured as 42.51 HU, and (**C**) the mean attenuation of the pons was measured as 22.40 HU. Arrows in panels (**B**,**C**) indicate the ROI boundaries. The relative CT number was calculated as 1.90.

Lesion attenuation was measured on five representative axial CT sections, selected to include the slice showing the largest cross-sectional area of the lesion and two adjacent slices above and below, using manually drawn freehand ROIs that encompassed the largest possible area of the solid component. Adjacent bony structures, air cavities, retained inflammatory secretions, areas of extensive necrosis, and regions susceptible to partial-volume effects at the lesion margins were carefully excluded from the ROIs. Although attenuation measurements represent mean voxel values within the ROI, differences in ROI area may influence tissue sampling and were therefore addressed in a sensitivity analysis. Pontine attenuation was similarly measured on five representative axial CT sections. The mean lesion and pontine attenuation values obtained from these measurements were used for subsequent analyses. The pons was selected as an internal reference region within the same examination to reduce the potential influence of acquisition- and reconstruction-related variability. Because absolute CT attenuation values may be influenced by scanner calibration, acquisition parameters, and image reconstruction settings, their direct comparability across patients and examinations may be limited. Therefore, lesion attenuation was normalized to pontine attenuation within the same examination. For each case, relative CT number was calculated as the ratio of mean lesion attenuation to mean pontine attenuation (relative CT number = HUlesion/HUpons) and was used as the primary quantitative imaging parameter (Figure 2, Figure 3 and Figure 4).

### 2.6. Assessment of Bone Changes

Bone changes adjacent to the lesion were evaluated on CT images by the same radiologist and classified into four categories: no bone change, bone thickening, bone erosion, and bone thinning. Bone thickening was defined as evident thickening of adjacent bony structures relative to the contralateral side; bone erosion as focal loss of cortical continuity; and bone thinning as marked thinning of the bony walls relative to the contralateral side.

### 2.7. Statistical Analysis

All statistical analyses were performed using IBM SPSS Statistics for Mac, version 26.0 (IBM Corp., Armonk, NY, USA). The distribution of continuous variables was assessed using visual methods and the Shapiro–Wilk test. Non-normally distributed continuous variables are presented as median [25th–75th percentile], and categorical variables as frequency and percentage (%). Differences in continuous variables across the SP, IP, and SCC groups were evaluated using the Kruskal–Wallis test, while categorical variables were compared using the chi-square test or Fisher’s exact test, as appropriate. For the predefined binary comparisons (SP vs. IP, IP vs. SCC, SP vs. IP+SCC, and SP vs. SCC), relative CT number values were compared using the Mann–Whitney U test. The diagnostic performance of relative CT number was assessed by receiver operating characteristic (ROC) curve analysis; the area under the curve (AUC), 95% confidence interval (CI), optimal cut-off value, sensitivity, and specificity were calculated.

Higher relative CT number values were considered positive test results. The positive outcome was IP for the SP versus IP comparison, SCC for the IP versus SCC and SP versus SCC comparisons and combined neoplastic lesions (IP+SCC) for the SP versus IP+SCC comparison. The optimal cut-off point was determined using the Youden index. The *p* value for each ROC analysis tested the null hypothesis that the AUC was equal to 0.5.

Exploratory thresholds achieving at least 90% sensitivity or at least 90% specificity were also identified from the ROC coordinates. Among the thresholds achieving at least 90% sensitivity, the cut-off with the highest corresponding specificity was selected; among those achieving at least 90% specificity, the cut-off with the highest corresponding sensitivity was selected.

Age-adjusted binary logistic regression analyses were performed for all four predefined binary comparisons. The same positive-outcome definitions used in the ROC analyses were applied to the logistic regression models. Age and relative CT number were entered simultaneously as independent variables. Odds ratios (ORs) were expressed per 0.1-unit increase in relative CT number and per 1-year increase in age and were reported with 95% confidence intervals.

For the IP–SCC comparison, additional exploratory analyses were performed to evaluate the influence of age. A model containing relative CT number alone was compared descriptively with the age-adjusted model. Potential effect modification by age was examined by adding an age × relative CT number interaction term to the model containing both main effects. Age and relative CT number were mean-centered within the IP–SCC subset before constructing the interaction term, and relative CT number was scaled in 0.1-unit increments. The interaction was evaluated using a likelihood ratio test comparing models with and without the interaction term. Collinearity between age and relative CT number in the main-effects model was assessed using variance inflation factors.

As an exploratory sensitivity analysis, lesion ROI area was added as a continuous covariate to each of the four age-adjusted logistic regression models. The resulting relative CT number estimates were compared with those from the corresponding models adjusted for age alone.

Exploratory logistic regression models containing age and relative CT number were compared with models additionally including bone changes as a four-level categorical variable. Differences in model fit were assessed using likelihood ratio tests, and discrimination was compared descriptively using apparent AUCs calculated in the same cohort without internal validation. Comparisons were reported where finite maximum-likelihood estimates could be obtained; models exhibiting quasi-complete separation were considered unsuitable for reliable estimation using standard logistic regression.

A two-sided *p*-value of less than 0.05 was considered statistically significant for all analyses. *p* values were reported without adjustment for multiple comparisons and should be interpreted as exploratory.

## 3. Results

A total of 92 patients were included in the study: 49 in the SP group, 24 in the IP group, and 19 in the SCC group. Demographic and clinicoradiological characteristics of the study groups are summarized in Table 1. A statistically significant difference in age was observed across the three groups (*p* < 0.001), with patients in the SCC group being older than those in the SP and IP groups. No significant differences were identified in sex distribution or side of lesion (*p* = 0.390 and *p* = 0.162, respectively).

**Figure 5:**
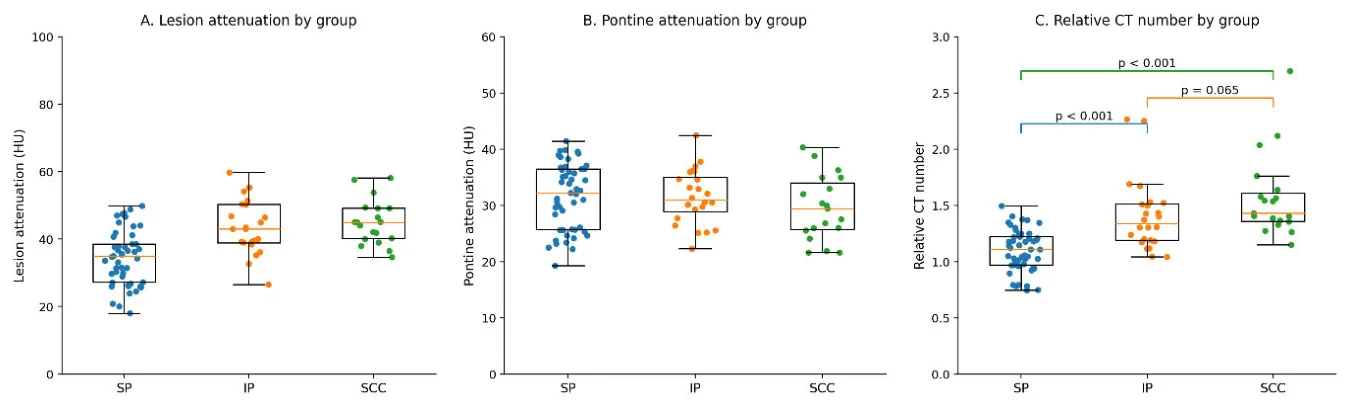
Lesion attenuation, pontine attenuation, and relative CT number across the study groups. (**A**) Distribution of lesion attenuation values in patients with SP, IP and SCC. (**B**) Distribution of pontine attenuation values used as the internal reference for normalization. (**C**) Distribution of relative CT number values, calculated as lesion attenuation divided by pontine attenuation, in the three study groups. Each dot represents an individual patient. Relative CT number was lowest in the SP group, intermediate in the IP group, and highest in the SCC group.

Relative CT number values differed significantly across the three groups (overall *p* < 0.001); median values were lowest in the SP group, intermediate in the IP group, and highest in the SCC group. Median relative CT number values were 1.107 [0.972–1.220] in the SP group, 1.339 [1.189–1.512] in the IP group, and 1.429 [1.359–1.607] in the SCC group (Figure 5). Lesion attenuation values also differed significantly across groups (*p* < 0.001). Lesion ROI area similarly showed a significant difference among the three groups (*p* = 0.001). In contrast, pontine attenuation values did not differ significantly across groups (*p* = 0.371), consistent with its use as an internal reference in this cohort.

In the SP versus IP comparison, relative CT number values were significantly higher in the IP group than in the SP group (*p* < 0.001). ROC analysis demonstrated good discriminative performance, with an AUC of 0.815 (95% CI, 0.704–0.904). The optimal cut-off value was 1.302, yielding a sensitivity of 62.5% and a specificity of 85.7% (Table 2).

There was no statistically significant difference in relative CT number between the IP and SCC groups in the unadjusted analysis (*p* = 0.065). Relative CT number showed limited standalone discriminative performance for differentiating IP from SCC (AUC, 0.667; 95% CI, 0.498–0.826). The 95% CI included 0.5, indicating that its standalone discrimination in this comparison could not be reliably distinguished from chance (Table 2).

In the SP versus combined neoplastic lesions (IP+SCC) comparison, relative CT number values were significantly higher in the combined neoplastic group than in the SP group (*p* < 0.001). ROC analysis yielded an AUC of 0.869 (95% CI, 0.792–0.932), with an optimal cut-off value of 1.262, a sensitivity of 76.7%, and a specificity of 81.6% (Table 2).

Among all predefined comparisons, the highest diagnostic performance was observed in the SP versus SCC comparison. Relative CT number values were markedly higher in the SCC group than in the SP group (*p* < 0.001), and ROC analysis demonstrated excellent discriminative performance, with an AUC of 0.937 (95% CI, 0.866–0.986), an optimal cut-off value of 1.262, a sensitivity of 94.7%, and a specificity of 81.6% (Table 2).

Exploratory sensitivity- and specificity-prioritized thresholds were also identified. All paired percentages below are reported as sensitivity/specificity. For SP versus IP, cut-offs of 1.116 and 1.371 yielded 91.7%/51.0% and 50.0%/93.9%, respectively. The corresponding thresholds were 1.262 and 1.761 for IP versus SCC (94.7%/37.5% and 21.1%/91.7%), 1.149 and 1.355 for SP versus IP+SCC (90.7%/57.1% and 62.8%/91.8%), and 1.262 and 1.355 for SP versus SCC (94.7%/81.6% and 78.9%/91.8%). These thresholds are exploratory and should not be interpreted as absolute diagnostic boundaries.

In age-adjusted binary logistic regression analyses, each 0.1-unit increase in relative CT number was associated with higher odds of IP rather than SP (OR, 2.04; 95% CI, 1.39–3.00; *p* < 0.001), SCC rather than IP (OR, 1.35; 95% CI, 1.03–1.77; *p* = 0.028), a neoplastic lesion rather than SP (OR, 2.30; 95% CI, 1.57–3.39; *p* < 0.001), and SCC rather than SP (OR, 5.99; 95% CI, 1.42–25.20; *p* = 0.015). The markedly wide confidence interval for this estimate reflects the limited sample size of the SCC group (n = 19) and indicates that this OR should be interpreted with caution rather than as a precise effect estimate.

Each 1-year increase in age was associated with higher odds of SCC rather than IP (OR, 1.10; 95% CI, 1.03–1.16; *p* = 0.002), a neoplastic lesion rather than SP (OR, 1.05; 95% CI, 1.02–1.09; *p* = 0.005), and SCC rather than SP (OR, 1.15; 95% CI, 1.04–1.28; *p* = 0.006), but not with IP rather than SP (OR, 1.03; 95% CI, 0.99–1.08; *p* = 0.100).

In the additional IP–SCC analyses (n = 43), the unadjusted OR per 0.1-unit increase in relative CT number was 1.16 (95% CI, 0.95–1.41; *p* = 0.144). The *p* value of 0.065 refers to the unadjusted Mann–Whitney U comparison, whereas *p* = 0.144 refers to the relative CT number coefficient in the unadjusted logistic regression model. Adding the age × relative CT number interaction term to the age-adjusted model did not significantly improve model fit (likelihood ratio test, *p* = 0.223). The variance inflation factor (VIF) was 1.04 for each predictor in the main-effects model, indicating no substantial collinearity between age and relative CT number.

In sensitivity analyses additionally adjusted for lesion ROI area, relative CT number remained associated with IP rather than SP (OR, 2.09; 95% CI, 1.39–3.12; *p* < 0.001), neoplastic lesions rather than SP (OR, 2.27; 95% CI, 1.53–3.38; *p* < 0.001), and SCC rather than SP (OR, 4.93; 95% CI, 1.20–20.21; *p* = 0.027). For SCC versus IP, the point estimate remained similar, but the confidence interval included 1 (OR, 1.34; 95% CI, 0.97–1.85; *p* = 0.073). All relative CT number ORs are expressed per 0.1-unit increase.

As a complementary qualitative imaging finding, a significant association was identified between bone changes and lesion type (*p* < 0.001). Absence of bone change was most frequently observed in the SP group (73.5%), whereas bone thickening was relatively more common in the IP group (29.2%) compared to the other groups. Bone erosion was the predominant pattern in the SCC group, occurring in 78.9% of cases, in contrast to only 4.1% in the SP group and 12.5% in the IP group.

Adding bone changes to the models containing age and relative CT number improved model fit for SP versus IP (likelihood ratio *p* = 0.005) and SP versus IP+SCC (*p* < 0.001). For SP versus IP, the apparent AUC increased from 0.840 for the age + relative CT number model to 0.907 after adding bone changes; for SP versus IP+SCC, it increased from 0.901 to 0.944. These AUCs were calculated in the same cohort and represent combined-model performance rather than the standalone relative CT number performance reported in Table 2. For IP versus SCC and SP versus SCC, the absence of SCC cases in some bone-change categories led to quasi-complete separation, preventing reliable estimation of the combined models.

## 4. Discussion

The present study evaluated the diagnostic performance of relative CT number for SP, IP, and SCC among patients with unilateral sinonasal lesions. Relative CT number differed significantly across the three groups overall, with median values lowest in SP, intermediate in IP, and highest in SCC. However, its discriminative performance varied across the predefined comparisons: it showed stronger performance for distinguishing SP from neoplastic lesions, particularly SCC, but more limited standalone performance for differentiating IP from SCC. These findings suggest that relative CT number may serve as an adjunctive quantitative CT parameter, particularly for differentiating inflammatory from neoplastic unilateral sinonasal lesions.

The findings of the present study regarding the SP versus IP comparison are largely consistent with those reported by Sano et al. [15], who evaluated 122 sinonasal masses and demonstrated that relative CT number was significantly higher in IP than in nasal polyps, with a threshold of >1.3 associated with IP on multivariate analysis, achieving a sensitivity of 72.6% and a specificity of 87.3%, and an AUC of 0.82. In our series, the AUC of 0.815 and the optimal cut-off of 1.302 for SP–IP discrimination were consistent with the values reported by Sano et al. [15]. The biological basis for this attenuation difference is consistent with established histopathological characteristics: SP typically exhibits edematous inflammatory stroma with a high water content, which corresponds to lower CT attenuation, whereas IP demonstrates dense epithelial proliferation and a more complex stromal architecture, resulting in higher attenuation values [15]. This histological contrast likely underlies the observed difference in relative CT number between the two lesion types.

The results of the IP versus SCC comparison partially corroborate the findings of Azuma et al. [14], who reported significantly higher relative CT number values in SCC compared to IP, with a threshold of ≥1.4 associated with SCC on multivariate analysis, achieving an AUC of 0.77, a sensitivity of 72%, and a specificity of 83%. In our series, although median relative CT number values were numerically higher in the SCC group, the difference did not reach statistical significance in the unadjusted analysis (*p* = 0.065), and the AUC of 0.667 reflected more modest discriminative performance. Several factors may account for this discrepancy. First, partial overlap in tissue density between IP and SCC likely reflects the histological heterogeneity of both lesion types, as previously noted in studies employing MRI-based diffusion-weighted imaging and apparent diffusion coefficient analysis for IP–SCC differentiation [16,17]. Second, the limited sample size of the SCC group in our series may have reduced statistical power specifically in this binary comparison. The difference between the unadjusted and age-adjusted findings warrants cautious interpretation, particularly given the substantial age imbalance and the small SCC subgroup. Additional analyses showed no substantial collinearity between age and relative CT number (VIF, 1.04) and no statistically significant age × relative CT number interaction (likelihood ratio test, *p* = 0.223). However, these findings do not exclude residual confounding by age or establish that the association is constant across ages, particularly given the limited sample size for assessing interaction. Although relative CT number was associated with SCC rather than IP in the main-effects age-adjusted model (OR, 1.35 per 0.1-unit increase; *p* = 0.028), this finding remains hypothesis-generating. The IP–SCC association was no longer statistically significant after additional adjustment for ROI area, despite little change in the point estimate, further supporting this cautious interpretation. Its potential contribution alongside other CT features [14,18] requires further evaluation before clinical recommendations can be made.

The higher relative CT number values observed in the SCC group are likely attributable to the distinct histopathological characteristics of malignant lesions. CT attenuation is known to reflect the physical density of tissue, which is in turn influenced by cellular composition, extracellular matrix content, vascular density, and water content [19]. Malignant tumors such as SCC typically exhibit increased cellularity, reduced stromal edema, and a more complex tissue architecture compared to both inflammatory polyps and benign neoplasms, all of which may contribute to higher CT attenuation values [7,19]. In contrast, SP, characterized by edematous inflammatory stroma with high water content, would be expected to demonstrate lower attenuation, consistent with our findings. IP, occupying an intermediate position histologically—with dense epithelial proliferation but without the full architectural complexity of malignancy—correspondingly shows intermediate relative CT number values. Taken together, the progressive attenuation gradient observed across the three groups may reflect an underlying biological continuum from inflammatory to benign neoplastic to malignant disease, providing a rational histopathological basis for the use of relative CT number as a quantitative discriminator in this spectrum [7,15].

A key advantage of relative CT number is that it can be readily obtained from routine non-contrast CT examinations without the need for advanced post-processing software or specialized computational infrastructure. While radiomics and deep learning approaches have demonstrated promising results in the characterization of sinonasal lesions [12,13,20], these methods typically require complex feature extraction pipelines, dedicated software platforms, and external validation before clinical deployment. In contrast, relative CT number requires only a freehand ROI measurement within the lesion and a corresponding pontine measurement on the same examination—a workflow that can be integrated into routine radiological reporting with minimal additional time or resources. This simplicity may make relative CT number a practical adjunct to morphological assessment, particularly in settings where advanced imaging tools are not readily accessible.

Normalization of lesion attenuation to pontine attenuation represents an important methodological feature of the relative CT number approach. Absolute HU values may vary according to CT acquisition and reconstruction parameters, limiting their direct comparability across examinations. In the present study, pontine attenuation was used as an internal reference within the same examination, with the intention of reducing the influence of acquisition- and reconstruction-related variability. Pontine attenuation did not differ significantly among the three diagnostic groups (*p* = 0.371), which was consistent with its use as an internal reference in this cohort. However, the absence of a between-group difference does not establish measurement stability or reproducibility, and cross-scanner, interobserver, and intraobserver reliability were not evaluated.

Fraczek et al. compared low-dose (120 kVp, 45 mAs) and standard-dose (120 kVp, 100 mAs) CT protocols. Their endoscopy study described noisier low-dose images, with greater image-quality deterioration in patients with extensive polyposis, supporting endoscopy-guided patient selection [11]. The anatomical assessment study reported poorer visualization of the lamina papyracea and cribriform plate in patients with advanced polyposis using low-dose CT [10]. Exposure time per rotation contributes to the tube current–time product (mAs); the low-dose and standard-dose protocols used rotation times of 0.4 and 0.7 s, respectively [10]. These studies compared acquisition protocols and did not isolate the effect of exposure time on image quality. Neither study evaluated relative CT number, and its applicability to reduced-dose protocols requires separate assessment.

Among the predefined comparisons, the most clinically relevant finding may be the strong discriminative performance of relative CT number in distinguishing SP from combined neoplastic lesions (AUC 0.869) and, most notably, from SCC specifically (AUC 0.937). These results suggest that relative CT number may have potential as an adjunct for identifying lesions warranting further workup. In clinical practice, a higher relative CT number, when considered alongside clinical and morphological findings, may contribute to the assessment of whether targeted endoscopic evaluation, biopsy, or further evaluation with MRI is warranted. Accordingly, relative CT number values exceeding the exploratory thresholds may increase suspicion for an underlying neoplastic process rather than an inflammatory lesion; however, these values should not be interpreted as disease-specific diagnostic boundaries, particularly because of the considerable overlap between IP and SCC. Therefore, relative CT number should not be used alone to determine the final diagnosis or treatment strategy.

In a retrospective analysis of unilateral paranasal sinus diseases, Lee et al. [8] emphasized that the combined assessment of patient age, clinical symptoms, and CT findings improves preoperative differential diagnosis, while also noting that bone erosion alone is not pathognomonic for malignancy. Our findings complement this perspective by demonstrating that a simple quantitative CT parameter—obtainable without additional imaging or post-processing—may provide complementary quantitative information alongside qualitative morphological assessment. The same Youden-derived optimal cut-off value of 1.262 was identified for both SP versus IP+SCC and SP versus SCC. This value may therefore represent a candidate threshold for prospective evaluation rather than an established clinical boundary. The sensitivity- and specificity-prioritized thresholds further illustrate the expected trade-off between identifying a greater proportion of neoplastic lesions and reducing false-positive classifications. Because all thresholds were derived from the same retrospective cohort, external validation is required before clinical application.

The bone change findings in the present study are consistent with previously reported patterns in the literature. Sano et al. [15] identified bone thickening as an independent discriminator between IP and SP on multivariate analysis, a finding corroborated by our data, in which bone thickening was relatively more prevalent in the IP group (29.2%) compared to SP (2.0%). Talati et al. [6] demonstrated that focal hyperostosis, while common in IP, is not universally present, and that absence of bone change does not exclude the diagnosis—a pattern reflected in our series, where 29.2% of IP cases showed no bone change. With respect to malignant lesions, a recent CT scoring system study by Umeda et al. [18] found that bone destruction and erosion at multiple sinonasal sites were significantly more frequent in SCC arising from IP compared to IP alone, supporting the role of bone erosion as a marker of malignant behavior. Consistent with this, bone erosion was the predominant pattern in our SCC group (78.9%), in contrast to only 4.1% in SP and 12.5% in IP. The observed differences in relative CT number and bone change patterns suggest that these parameters may reflect distinct aspects of lesion assessment. Exploratory analyses showed that adding bone changes to age and relative CT number improved model fit for SP versus IP and SP versus IP+SCC. These findings suggest that considering lesion attenuation together with bone changes may aid the distinction between inflammatory and neoplastic lesions. However, the apparent AUCs were estimated in the same cohort without internal validation, and quasi-complete separation prevented reliable estimation of the IP–SCC and SP–SCC combined models.

The clinical significance of IP extends well beyond its histologically benign classification. IP is associated with a high recurrence rate following surgical resection and carries a well-documented risk of malignant transformation to SCC, with reported rates ranging from 5% to 13% in the literature [5,14,16]. When malignant transformation occurs, prognosis deteriorates substantially; reported 5-year overall survival rates for SCC arising from IP range from approximately 40% to 56%, underscoring the critical importance of early and accurate preoperative identification [21]. These prognostic implications directly influence surgical strategy: while SP can often be managed with limited endoscopic intervention, IP requires wider resection of involved mucosa and underlying bone to minimize recurrence risk, and SCC necessitates even more aggressive resection, potentially combined with adjuvant radiotherapy or chemotherapy [6,14]. Accurate preoperative differentiation across this pathological spectrum has potential consequences for the extent of surgery and the need for adjuvant treatment. Our findings suggest that the principal potential role of relative CT number lies in the initial differentiation of inflammatory from neoplastic lesions. Its contribution to IP–SCC differentiation appears more limited and should be considered together with patient age, bone change patterns, endoscopic findings, and, where clinically indicated, MRI features such as diffusion-weighted imaging [16,17].

Several limitations of the present study warrant acknowledgment. First, the retrospective single-center design introduces the possibility of selection bias and limits generalizability. All examinations were obtained using a single CT scanner and an institutional acquisition protocol; therefore, the performance of relative CT number across different scanners, dose settings, and reconstruction protocols was not evaluated. Although normalization to pontine attenuation was intended to reduce acquisition-related variability, this study does not demonstrate scanner or protocol invariance. The absence of a significant difference in pontine attenuation among the diagnostic groups does not establish stability across acquisition settings. Consequently, the transferability of the reported diagnostic performance and cut-off values requires validation using data from other scanners and institutions. Second, the limited sample size and unequal group sizes (SP, n = 49; IP, n = 24; SCC, n = 19) limit the precision of estimates involving SCC and warrant caution when interpreting the adjusted analyses. For IP versus SCC, standalone discrimination was modest (AUC, 0.667; 95% CI, 0.498–0.826), with the confidence interval including 0.5. Although an association was observed after adjustment for age, this finding should be regarded as hypothesis-generating. The wide confidence interval for the adjusted SP–SCC odds ratio (OR, 5.99; 95% CI, 1.42–25.20) also indicates substantial uncertainty in the magnitude of that association. A formal a priori sample size calculation was not performed. Larger independent cohorts are needed to obtain more precise estimates and assess the robustness of these findings. Third, measurements were initially performed by one radiologist and subsequently reviewed jointly by both radiologists, with final measurements agreed upon by consensus. Independent readings and repeated measurements were not recorded; therefore, formal inter- and intra-observer reproducibility could not be assessed using the available measurements. Future studies should include independent blinded readings and repeated measurements to assess reproducibility. Fourth, the present analysis was confined to quantitative measurements derived from non-contrast CT; MRI features—including diffusion-weighted imaging and apparent diffusion coefficient values, which have shown promise in differentiating IP from IP-associated SCC [16,17]—were not incorporated, nor were advanced radiomics or deep learning parameters [12,13,20]. Fifth, the direct correlation between relative CT number and specific histopathological characteristics, such as cellular density, stromal composition, and degree of keratinization, was not quantitatively examined, which limits mechanistic interpretation of the observed attenuation differences. Sixth, lesion ROI area differed significantly across the three groups. Although this was addressed in a sensitivity analysis, adjustment for ROI area cannot fully account for differences in tissue heterogeneity or sampling. The expanded models should also be interpreted cautiously given the limited sample size, particularly in the SCC subgroup. Seventh, sparse bone-change categories prevented reliable estimation of the combined models for the IP–SCC and SP–SCC comparisons. The low number of events per estimated parameter in the combined models increased the risk of overfitting. The performance of the estimable models was assessed in the same cohort without internal validation and may therefore be optimistic. Finally, disease stage and the distinction between de novo and IP-associated SCC were not systematically recorded, precluding assessment of their potential influence on relative CT number.

## 5. Conclusions

In conclusion, relative CT number, a quantitative parameter readily obtainable from routine non-contrast CT, showed promising diagnostic performance in differentiating inflammatory from neoplastic unilateral sinonasal lesions in this retrospective single-center cohort. The highest discrimination was observed for SP versus SCC (AUC, 0.937), followed by SP versus combined neoplastic lesions (AUC, 0.869), suggesting a potential role as an adjunct to morphological CT assessment. In contrast, standalone discrimination between IP and SCC was limited. Although relative CT number was associated with SCC rather than IP after adjustment for age, this association was not robust to additional adjustment for ROI area. This finding should therefore be considered hypothesis-generating rather than evidence of established clinical utility. The small SCC subgroup and unequal group sizes limit the precision of the estimates and warrant caution when interpreting the adjusted associations. The cut-off values reported here should be regarded as exploratory rather than validated clinical thresholds. Our exploratory findings suggest that adding bone changes to age and relative CT number may improve differentiation of SP from IP and from combined neoplastic lesions. Larger prospective multicenter studies are needed to validate this potential added value, assess measurement reproducibility, and evaluate the contribution of other clinical and MRI features.

## Figures and Tables

**Table 1:** Demographic and clinicoradiologic characteristics of the study groups.

Variable	SP (n = 49)	IP (n = 24)	SCC (n = 19)	*p* Value
Age, years	50.0 [37.0–59.0]	57.5 [50.8–64.0]	74.0 [61.0–85.0]	<0.001
Sex				0.390
Male	29 (59.2)	18 (75.0)	13 (68.4)	
Female	20 (40.8)	6 (25.0)	6 (31.6)	
Lesion side				0.162
Right	24 (49.0)	16 (66.7)	8 (42.1)	
Left	25 (51.0)	7 (29.2)	11 (57.9)	
Choana	0 (0.0)	1 (4.2)	0 (0.0)	
Lesion attenuation, HU	35.0 [27.3–38.6]	43.0 [38.9–50.3]	44.9 [40.1–49.2]	<0.001
Lesion ROI area, mm^2^	209.5 [99.6–283.6]	240.9 [153.9–294.0]	312.0 [239.5–634.0]	0.001
Pontine attenuation, HU	32.2 [25.6–36.5]	31.0 [28.9–35.0]	29.4 [25.7–34.0]	0.371
Relative CT number	1.107 [0.972–1.220]	1.339 [1.189–1.512]	1.429 [1.359–1.607]	<0.001
Bone change				<0.001
None	36 (73.5)	7 (29.2)	0 (0.0)	
Thickening	1 (2.0)	7 (29.2)	0 (0.0)	
Erosion	2 (4.1)	3 (12.5)	15 (78.9)	
Thinning	10 (20.4)	7 (29.2)	4 (21.1)	

Data are presented as median [25th–75th percentile] or n (%). Abbreviations: SP, sinonasal polyp; IP, inverted papilloma; SCC, squamous cell carcinoma; HU, Hounsfield unit; ROI, region of interest. *p* values represent overall comparisons among the SP, IP, and SCC groups and were calculated using the Kruskal–Wallis test for continuous variables and the chi-square test or Fisher’s exact test for categorical variables, as appropriate. Pairwise comparisons are not presented in this table.

**Table 2:** ROC analysis of relative CT number for the predefined binary diagnostic comparisons.

Comparison	AUC (95% CI)	Optimal Cut-Off	Sensitivity (%)	Specificity (%)	*p* Value
SP vs. IP	0.815 (0.704–0.904)	1.302	62.5	85.7	<0.001
IP vs. SCC	0.667 (0.498–0.826)	1.327	84.2	50.0	0.065
SP vs. IP+SCC	0.869 (0.792–0.932)	1.262	76.7	81.6	<0.001
SP vs. SCC	0.937 (0.866–0.986)	1.262	94.7	81.6	<0.001

Abbreviations: AUC, area under the curve; CI, confidence interval; SP, sinonasal polyp; IP, inverted papilloma; SCC, squamous cell carcinoma. Optimal cut-off values were determined using the Youden index. Cut-offs are exploratory and unvalidated; performance may be optimistic because thresholds were derived and evaluated in the same cohort. *p* values are unadjusted for multiple comparisons.

## Data Availability

The data presented in this study are available on request from the corresponding author due to restrictions. The data are not publicly available due to patient privacy concerns and ethical restrictions, in accordance with the approval of the institutional ethics committee and the Turkish Personal Data Protection Law (Law No. 6698).

## Abbreviations

The following abbreviations are used in this manuscript:

CT	Computed Tomography
SP	Sinonasal Polyp
IP	Inverted Papilloma
SCC	Squamous Cell Carcinoma
HU	Hounsfield Unit
ROI	Region of Interest
ROC	Receiver Operating Characteristic
AUC	Area Under the Curve
IRB	Institutional Review Board
MRI	Magnetic Resonance Imaging
CI	Confidence Interval
OR	Odds Ratio
VIF	Variance Inflation Factor
